# Hydropneumothorax as a complication of necrotizing pneumonia in a young girl

**DOI:** 10.1002/ccr3.2294

**Published:** 2019-07-08

**Authors:** Jamison Cole Miller, Thomas George Boyce

**Affiliations:** ^1^ Levine Children's Hospital Charlotte North Carolina

**Keywords:** bronchopleural fistula, hydropneumothorax, pediatric medicine, pneumonia

## Abstract

Bronchopleural fistula with subsequent hydropneumothorax is an important complication of necrotizing pneumonia. Chest X‐ray is an excellent diagnostic tool which can suggest hydropneumothorax. When present, this requires admission for drainage. If discharged after necrotizing pneumonia, follow‐up should include a chest X‐ray to rule out this complication.

## 1 CASE

A 5‐year‐old previously healthy girl presented to the hospital with five days of fever up to 104.1 F, myalgia, cough, congestion, and post‐tussive emesis. Additionally, she had decreased appetite, dyspnea, and left‐sided chest pain prompting admission. Three days before presentation, a rapid test for influenza A was positive, and she was started on oseltamivir. She had been born full term without complications. Other than asthma in her father, family history was noncontributory. She had never been hospitalized nor had any surgical history. Her vaccinations were up‐to‐date with the exception of the influenza vaccine.

Vital signs were notable for a heart rate of 152 beats per minute, respiratory rate of 42 breathes per minute on 1 L of oxygen by nasal cannula with saturations of 95% and blood pressure of 115/80. On examination, she was sleeping but aroused to stimulation. Perfusion was normal but she had dry mucus membranes and tachycardia. She was tachypneic with intercostal and supraclavicular retractions. Her breathing was noted as shallow with intermittent grunting. Breath sounds at the left lung zones, particularly the base, were diminished.

Laboratory findings demonstrated a white blood cell count of 6.3 per mcL [6.3 per 10^9 ^L] with 15% bands and 56% neutrophils, hemoglobin of 13.4 g/dL [8.3 mmol/L], and platelet count of 276 per mcL [276 per 10^9 ^L]. C‐reactive protein was 15.3 mg/dL [1457 nmol/L] with a sedimentation rate of 88 mm/h.

A chest X‐ray was obtained showing opacification of the left lower lobe with a possible left‐sided pleural effusion. Ampicillin‐sulbactam was initiated given concern for complicated pneumonia with parapneumonic effusion. Chest ultrasound further revealed multiple internal septations and debris within the effusion. Pediatric surgery and infectious disease were consulted, and antibiotics were transitioned to ceftriaxone and clindamycin. Video‐assisted thoracoscopic surgery (VATS) was performed with decortication and evacuation of an empyema followed by placement of a left‐sided chest tube. The culture from the empyema revealed no growth. After decreasing chest tube output, it was removed.

**Figure 1:**
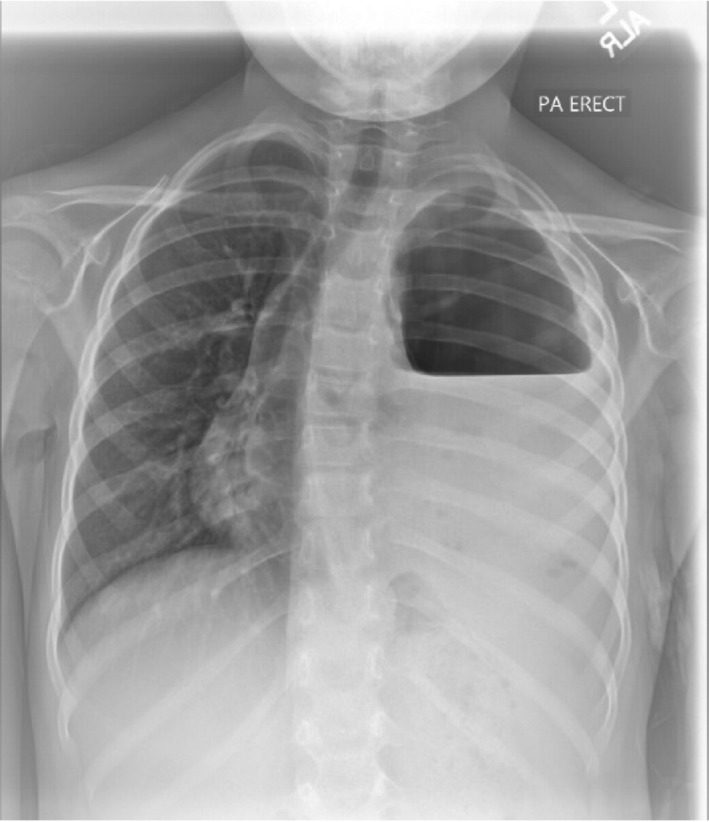
Chest X‐ray showing left‐sided pleural air‐fluid level with mediastinal shift concerning for hydropneumothorax

**Figure 2:**
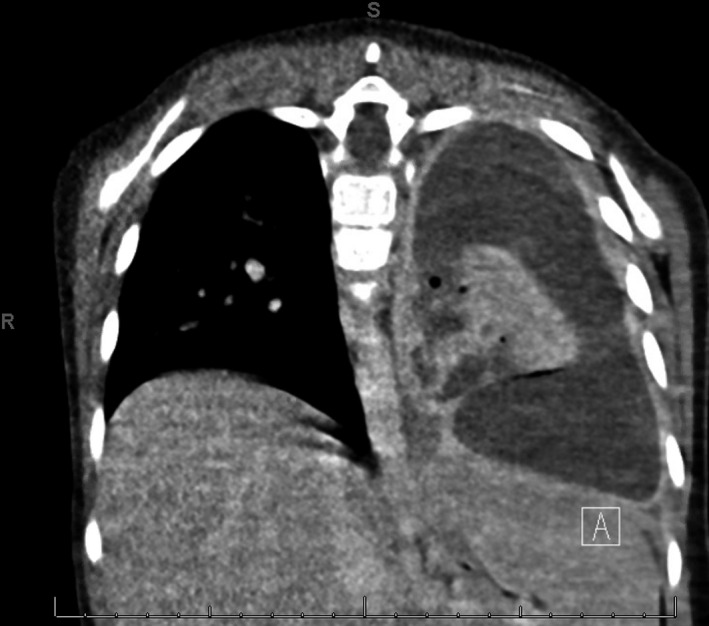
Chest CT coronal view demonstrating left lower lung necrosis with atelectasis and extensive effusion

The next day a chest X‐ray showed an air‐fluid level in the left pleural space with rightward mediastinal shift, suspicious for a hydropneumothorax (Figure 1). The following day a chest CT without contrast found a moderately large, complex left hydropneumothorax, containing an air‐fluid level with rightward shift of the mediastinal structures, left lower lobe necrosis, and compression of the entire left lung (Figure 2). A chest tube was placed under ultrasound guidance to allow drainage. Two days later, alteplase was administered over three days without improvement. Following this treatment failure without adequate lung expansion, VATS was again performed with removal of purulent exudate and necrotic portions of the left lower lobe. After six days, patient was able to ambulate. Chest films for the next week demonstrated an air leak that did not allow expansion of the left lung. Two weeks following, the air leak had resolved with no evidence of residual left hydropneumothorax and continued success in toleration of oral feeding and ambulation. Patient was then discharged home and completed 6 weeks of intravenous ceftriaxone and clindamycin.

## 2 DISCUSSION

Our patient developed a hydropneumothorax likely due to a bronchopleural fistula (BPF) formed from her extensive necrotizing pneumonia. Hydropneumothorax occurs when free fluid and air enters the pleural space. While rare, it has been associated with malignancy, infection, chest trauma, rheumatologic diseases that affect lung parenchyma, following chest tube placement, and after a thoracentesis.^1^ As seen in our case, patients can develop hydropneumothorax due to BPF secondary to a complicated pneumonia. To the authors’ knowledge, necrotizing pneumonia with secondary bronchopleural fistula and hydropneumothorax is such a rare entity that an incidence has not been reported.

Bronchopleural fistula and hydropneumothorax are most commonly reported secondary to staphylococcal and pneumococcal pneumonia, especially when the pneumonia is necrotizing. Breakdown of lung parenchyma allows a disruption in the bronchial wall and subsequent communication with the pleural space. On inspiration, air flows through the respiratory passages and enters the pleural space. The bronchial tear can produce a valve‐like effect which allows air entry into the pleural space during inspiration but traps it during expiration.

Presenting signs and symptoms of hydropneumothorax include sudden shortness of breath and localized chest pain. Decreased chest excursion, asymmetric breath sounds, and poor air entry can be notable on examination. If hydropneumothorax is large, shifting of mediastinal structures occurs with movement of the cardiac point of maximal impulse and tracheal deviation. Hypotension may develop as vena cava compression and increased intrathoracic pressure negatively impact venous return and stroke volume.

Diagnosis of hydropneumothorax is confirmed following a chest CT that reveals free air and fluid levels within the pleural space with collapsed lung parenchyma. A chest X‐ray can reveal a sharp pleural line with an adjacent opacity and elevation of the hemidiaphragm with mediastinal shift.

Bronchopleural fistula can be an evasive diagnosis. Typically, this requires multiple images and bronchoscopies. Non‐contrast computed tomography and 3‐dimensional spiral reconstruction both can aid in finding the BPF.^2^, ^3^ Fiberoptic bronchoscopy and selective bronchography can definitively identify fistula location.^2^ Despite these measures, definitive demonstration of the BPF is often lacking. Management is often based on suspected BPF given the clinical and radiographic presentation.

Initial management should include assessing the airway and breathing while placing cardiac monitors to determine the need for immediate stabilization. Distinguishing a hydropneumothorax from a simple pleural effusion is necessary because treatment of hydropneumothorax frequently requires placement of two chest tubes, one within the fluid and one to remove the air.^1^ Surgical management is an option for BPF in pediatric empyema and necrotizing pneumonia. A retrospective study found that early surgery featuring limited decortication and insertion of the serratus anterior muscle digitation flap to be safe and avoid morbidity associated with conservative management and necrotic lung resection surgery.^4^ In other cases of small fistula or when surgical risk is high, bronchoscopic techniques are preferable to close the fistula.^5^

## 3 CONCLUSION

In summary, BPF with subsequent hydropneumothorax is an uncommon but important complication of necrotizing pneumonia. Patients who are discharged from the hospital after treatment for a complicated pneumonia should have a chest X‐ray performed in the first week as an outpatient to detect the presence of air and fluid in the pleural space. If present, they should be emergently readmitted to the hospital for drainage of the hydropneumothorax.

## CONFLICT OF INTEREST

None declared.

## AUTHOR CONTRIBUTIONS

JCM: Corresponding author and pediatric resident responsible for manuscript production, care of the patient, literature review, and image submission. TGB: Attending pediatric infectious disease physician responsible for helping to make significant edits to manuscript, providing guidance for publication and manuscript outline, primary doctor of the patient, literature review, and image understanding.

## References

[1] Ortega C , Gonzales C , Soto‐Martinez ME , Yock‐Corrales A . Hydropneumothorax in children: a rare complication of a bacterial pneumonia. Case Rep Pediatr. 2016;2016:1‐4. 10.1155/2016/8097105 PMC4876213 27247819

[2] Westcott JL , Volpe JP . Peripheral bronchopleural fistula: CT evaluation in 20 patients with pneumonia, empyema, or postoperative air leak. Radiology. 1995;196:175‐181. 7784563 10.1148/radiology.196.1.7784563

[3] Vogel N , Wolcke B , Kauczor HU , Kelbel C , Mildenberger P . Detection of a bronchopleural fistula with spiral CT and 3D reconstruction. Aktuelle Radiol. 1995;5:176‐178. 7605817

[4] York EL , Lewall DB , Hirji M , Gelfand ET , Modry DL . Endoscopic diagnosis and treatment of postoperative bronchopleural fistula. Chest. 1990;97:1390‐1392. 2347224 10.1378/chest.97.6.1390

[5] Sarkar P , Chandak T , Shah R , Talwar A . Diagnosis and management of bronchopleural fistula. Indian J Chest Dis Allied Sci. 2010;52:97‐104. 20578402

